# Tertiary lymphoid structures as a potential prognostic biomarker for combined hepatocellular–cholangiocarcinoma

**DOI:** 10.1007/s12072-024-10694-2

**Published:** 2024-05-20

**Authors:** Wenchen Gong, Su Zhang, Xiangdong Tian, Wenshuai Chen, Yuchao He, Liwei Chen, Tingting Ding, Peiqi Ren, Lin Shi, Qiang Wu, Yan Sun, Lu Chen, Hua Guo

**Affiliations:** 1 Department of Tumor Cell Biology, Tianjin Medical University Cancer Institute and Hospital, Tianjin, 300060 China; 2 Department of Pathology, Tianjin Medical University Cancer Institute and Hospital, Tianjin, 300060 China; 3 Department of Gynecological Oncology, Tianjin Medical University Cancer Institute and Hospital, Tianjin, 300060 China; 4 Department of Endoscopic Diagnosis and Therapy, Tianjin Medical University Cancer Institute and Hospital, Tianjin, 300060 China; 5 Department of Hepatobiliary Cancer, Tianjin Medical University Cancer Institute and Hospital, Liver Cancer Research Center, Tianjin, 300060 China; 6 National Center for Global Health and Medicine, Department of Hepato-Biliary-Pancreatic Surgery, Tokyo, Japan; 7 National Clinical Research Center for Cancer, National Key Laboratory of Druggability Evaluation and Systematic Translational Medicine, Tianjin Key Laboratory of Digestive Cancer, Tianjin’s Clinical Research Center for Cancer, Tianjin, 300060 China

**Keywords:** Combined hepatocellular–cholangiocarcinoma, Tertiary lymphoid structures, Prognosis, Immunotherapy response, CXCL12

## Abstract

**Background:**

Combined hepatocellular–cholangiocarcinoma (cHCC–CCA), as a rare primary hepatic tumor, is challenging to accurately assess in terms of the clinical outcomes and prognostic risk factors in patients. This study aimed to clarify the function of tertiary lymphoid structure (TLS) status in predicting the outcome of cHCC–CCA and to preliminarily explore the possible mechanism of TLS formation.

**Methods:**

The TLSs, with different spatial distributions and densities, of 137 cHCC–CCA were quantified, and their association with prognosis was assessed by Cox regression and Kaplan–Meier analyses. We further validated TLS possible efficacy in predicting immunotherapy responsiveness in two cHCC–CCA case reports. TLS composition and its relationship to CXCL12 expression were analysed by fluorescent multiplex immunohistochemistry.

**Results:**

A high intratumoural TLS score was correlated with prolonged survival, whereas a high TLS density in adjacent tissue indicated a worse prognosis in cHCC–CCA. Mature TLSs were related to favorable outcomes and showed more CD8 + T cells infiltrating tumor tissues. We further divided the cHCC–CCA patients into four immune grades by combining the peri-TLS and intra-TLS, and these grades were an independent prognostic factor. In addition, our reported cases suggested a potential value of TLS in predicting immunotherapy response in cHCC–CCA patients. Our findings suggested that CXCL12 expression in cHCC–CCA tissue was significantly correlated with TLS presence.

**Conclusion:**

The spatial distribution and density of TLSs revealing the characteristics of the cHCC–CCA immune microenvironment, significantly correlated with prognosis and provided a potential immunotherapy response biomarker for cHCC–CCA.

**Supplementary Information:**

The online version contains supplementary material available at 10.1007/s12072-024-10694-2.

## Introduction

Combined hepatocellular–cholangiocarcinoma (cHCC–CCA) is a rare type of primary liver cancer (PLC) characterized by both hepatocellular and biliary epithelial phenotypes and has an incidence that varies between 0.4% and 14.2% [1]. cHCC–CCA, as a highly heterogeneous tumor, is reported to have a more aggressive behavior and poorer prognosis than hepatocellular carcinoma (HCC) and intrahepatic cholangiocarcinoma (ICC) [2]. Although the diagnostic and treatment methods have improved in recent years, the effect of traditional therapeutic strategies on improving the prognosis of cHCC–CCA is negligible [3]. The recent success of immunotherapy in various solid malignancies brings new opportunities to cHCC–CCA patients, but the effect on antitumoural responses needs to be better understood and evaluated. Due to the rarity of cases and the difficulty of diagnosis, accurate survival prediction, high-risk patient screening, and personalized treatment remain to be further elucidated.

Tertiary lymphoid structures (TLSs), referred to as heterotopic lymphoid tissues, are lymphoid tissues with a structure highly similar to that of secondary lymphoid organs (SLOs) [4]. TLSs occur in nonlymphoid tissues with pathological states, such as chronic infection, organ transplantation, autoimmune diseases, and cancer [5]. Several recent studies have shown that TLSs exist in various tumors, including melanoma, breast cancer, lung cancer, colorectal cancer, and pancreatic cancer, and are related to a favorable prognosis [6]. Due to the lack of an outer fibrous wrapping, the aggregated immune cells of TLSs are widely exposed to the tumor microenvironment (TME), leading to stimulation by tumor antigens and cytokines, becoming more direct and extensive sites for an antitumor immune response. Their presence is associated with a lower risk of recurrence and improved survival in virtually all solid tumors [7]. TLSs comprise a T-cell area, a B-cell follicular area, dendritic cells, and scattered high endothelial veins and are divided into mature and immature TLSs according to whether they have a germinal centre (GC) structure [4]. TLS formation, in terms of the quantity and density, indicates that tumor antigens are recognized by the immune system. As a lymphoid organ-like structure, TLSs provide a structural and functional basis for tumor-infiltrating lymphocytes in the TME [6]. Considerable heterogeneity of TLSs exists in cancer types, their location within tumors, and different patients [8]. Previous studies have shown that TLSs can be used to predict the prognosis of patients with HCC or ICC; however, their function and significance in PLCs are still controversial [9, 10]. Moreover, recent studies have successively demonstrated that TLS formation is associated with a favourable response to immunotherapy in melanoma and sarcoma [11, 12]. Accordingly, the significance of TLSs in predicting the prognosis and immunotherapy response in cHCC–CCA warrants investigation.

The formation of TLSs is mainly due to the occurrence of extranodal inoculation of lymphoid tissue caused by persistent chronic inflammation, and a set of chemokine ligands contribute to regulating the recruitment of immune cells to lymphoniches [13]. However, the underlying mechanism of TLS formation remains ambiguous. Some studies have shown that tissue-specific expression of SLO-related chemokines such as CXCL13, CCL21, CCL19, and CXCL12 can promote the formation of local TLSs in mouse models [14]. Antigen recognition-inducing immune responses contribute to TLS formation [6]. tumors, as tissues that escape immune surveillance, secrete chemokines that may play a notable role in the formation of TLSs. CXCL12 can be released by injured hepatocytes, biliary epithelial cells, sinusoidal endothelial cells, tumor-associated leukocytes, and HCC cells themselves and have proinflammatory, profibrotic, and proangiogenic functions [15]. A recent study demonstrated that CXCL12 plays a crucial role in regulating GC B-cell trafficking and GC formation [16]. However, the function of CXCL12 in PLC patients remains elusive [17, 18]. The aim of this study was to investigate TLS function and its relationship with CXCL12 in cHCC–CCA and to clarify the distribution, constituents, and prognostic value of TLSs in the cHCC–CCA tissues of 137 patients, hoping to provide some insight into the accurate stratification, prognosis prediction, and treatment of patients with cHCC–CCA.

## Materials and methods

### Patients and tissue samples

The discovery cohort consisted of 137 patients who underwent surgical resection for cHCC–CCA at Tianjin Medical University Cancer Institute and Hospital from January 2009 to December 2020. None of the patients received any molecular-targeted or immune therapy before surgery or during the follow-up period. The exclusion criteria were palliative resection, transarterial embolization, incomplete survival information, and distant metastasis. All hematoxylin–eosin (H&E) and immunohistochemistry sections of the patients were independently reviewed by two senior pathologists in a double-blinded manner. According to the International Classification of Diseases for Oncology, all cases were diagnosed as cHCC–CCA: ICD-O code 8180/3. CHCC–CCA is defined by the unequivocal presence of both hepatocellular and cholangiocytic differentiation within the same tumor. The two components show all the architectural and cytological differentiation patterns described for HCC and CCA, respectively. The immunohistochemistry of HCC shows positive staining for Hepatocyte and Glypican 3; CCA shows positive staining for CK7 and CK19 [19]. This work was performed in accordance with the requirements of the Declaration of Helsinki and was approved by the ethics committee of Tianjin Medical University Cancer Institute and Hospital.

### Immunohistochemical (IHC) staining

Consecutive serial sections of formalin-fixed paraffin-embedded (FFPE) tissue were prepared and processed on a Ventana BenchMark XT apparatus (Ventana Medical Systems). The sections were dewaxed, followed by antigen repair at 95 °C for 30 min in EDTA repair solution. After incubation with primary antibodies against CD4 (ZM0418, Zhongshan Chemical Co., Beijing, China), CD8 (SP57, Ventana Medical Systems, Tucson, AZ, USA), CD20 (L26, Ventana, Tucson, AZ, USA), CD21 (ZA0525, Zhongshan Chemical Co., Beijing, China), CD23 (ZM0273, Zhongshan Chemical Co., Beijing, China), CXCL12 (BS4938R, Bioss, Beijing, China), CK19 (ZM0670, Zhongshan Chemical Co., Beijing, China), Glypican-3 (ZM0146, Zhongshan Chemical Co., Beijing, China), programmed death 1 (PD-1) (ZM0381, Zhongshan Chemical Co., Beijing, China), and programmed death ligand 1 (PD-L1) (SP263, Ventana, Tucson, AZ, USA) at 37 °C for 32 min, the sections were then incubated with an HRP‐conjugated secondary antibody (multimer HRP, Ventana) for 10 min at room temperature. The positive signal was visualized with diaminobenzidine, followed by counterstaining with haematoxylin.

### IHC analysis

The degree of IHC staining was assessed by a staining index (SI, sum of staining intensity and percentage), which was semiquantitatively evaluated by two pathologists. Ten randomly selected fields from each section at 400 × magnification were analysed, and CXCL12 staining was located in the cell membrane and cytoplasm. The staining percentage was classified into four categories: no significant area, score of 0; positive cell rate ≤ 25%, score of 1; positive cell rate 25–50%, score of 2; and positive cell rate > 50%, score of 3. The staining intensity was classified into four grades: grade 0 (negative staining), grade 1 (light staining), grade 2 (intermediate staining), and grade 3 (intense staining). An SI score ≥ 3 was identified as high expression, while an SI score < 3 was identified as low expression. In addition, IHC staining confirmed the existence and structure of TLSs: CD21 and CD23 marked the GC, CD20 marked the B-cell area, and CD4 and CD8 marked the T-cell area.

### TLS definition and quantification

All H&E-stained sections of each patient were evaluated for the presence and location of TLSs by two pathologists blinded to the clinicopathological parameters of the patients. TLSs were classified as follows: lymphocyte aggregation: vague and ambiguous lymphocyte clusters; primary lymphoid follicles: round lymphocyte clusters without GCs; and secondary lymphoid follicles: GCs in round lymphocyte clusters. In addition, according to whether a GC was present, TLSs were divided into immature TLSs (including lymphoid aggregates and primary lymphoid follicles) and mature TLSs (secondary lymphoid follicles). Finally, further grading was carried out according to the number and density of TLSs.

### Immune cell infiltration analysis of TCGA and GEO data

The mRNA-seq and clinical information of the TCGA liver hepatocellular carcinoma (LIHC) cohort were obtained from the official websites. To investigate the correlation between CXCL12 expression and the abundance of diverse immune cell types, we employed the "psych" package in R by combining the official CIBERSORT source code (https://cibersortx.stanford.edu/) with the LM22.txt as reference database. We obtained microarray gene expression data (Affymetrix Human Exon 1.0 ST Array) from formalin-fixed, paraffin-embedded (FFPE) samples with 115 HCC patients’ cohort from Gene Expression Omnibus (GSE76427) using the GEO query R package (v. 2.54.1).

### Fluorescent multiplex immunohistochemistry

Consecutive 3-µm-thick slices were cut from FFPE cHCC–CCA tissues, followed by heat-induced antigen retrieval with an EDTA-based buffer for 10 min. The primary antibodies (including antibodies against CD20, CD21, CD4, CD8, and CXCL12) were added to each section and incubated at 4 °C overnight. After incubation with the secondary antibody, the corresponding HRP-binding fluorescent dyes were applied to each antibody in the following order: Opal-480, Opal-520, Opal-570, Opal-620, and Opal-690. Finally, after conducting thermal repair again, DAPI was used to stain the nucleus. An anti-fluorescence quenching agent was added, and the slides were examined using a fluorescence microscope.

### Statistical analysis

Statistical analysis was performed using SPSS 26.0 software (SPSS Inc., Chicago, USA). The IHC score of paraffin tissue samples and its relationship with TLSs were analysed by Pearson’s Chi-square test or Spearman’s test. Kaplan‒Meier survival analysis was conducted to analyse the relationship between TLS grade and disease recurrence or overall survival (OS) in cHCC–CCA patients. The log-rank test was used to detect the difference between survival curves. Univariate analysis was used to evaluate the prognostic factors of OS, and the factors found to be significant in the univariate analysis were subjected to multivariate regression analysis. A two-tailed *p* < 0.05 was considered statistically significant.

## Results

### Clinicopathological characteristics of cHCC–CCA patients and TLS identification

**Fig. 1:**
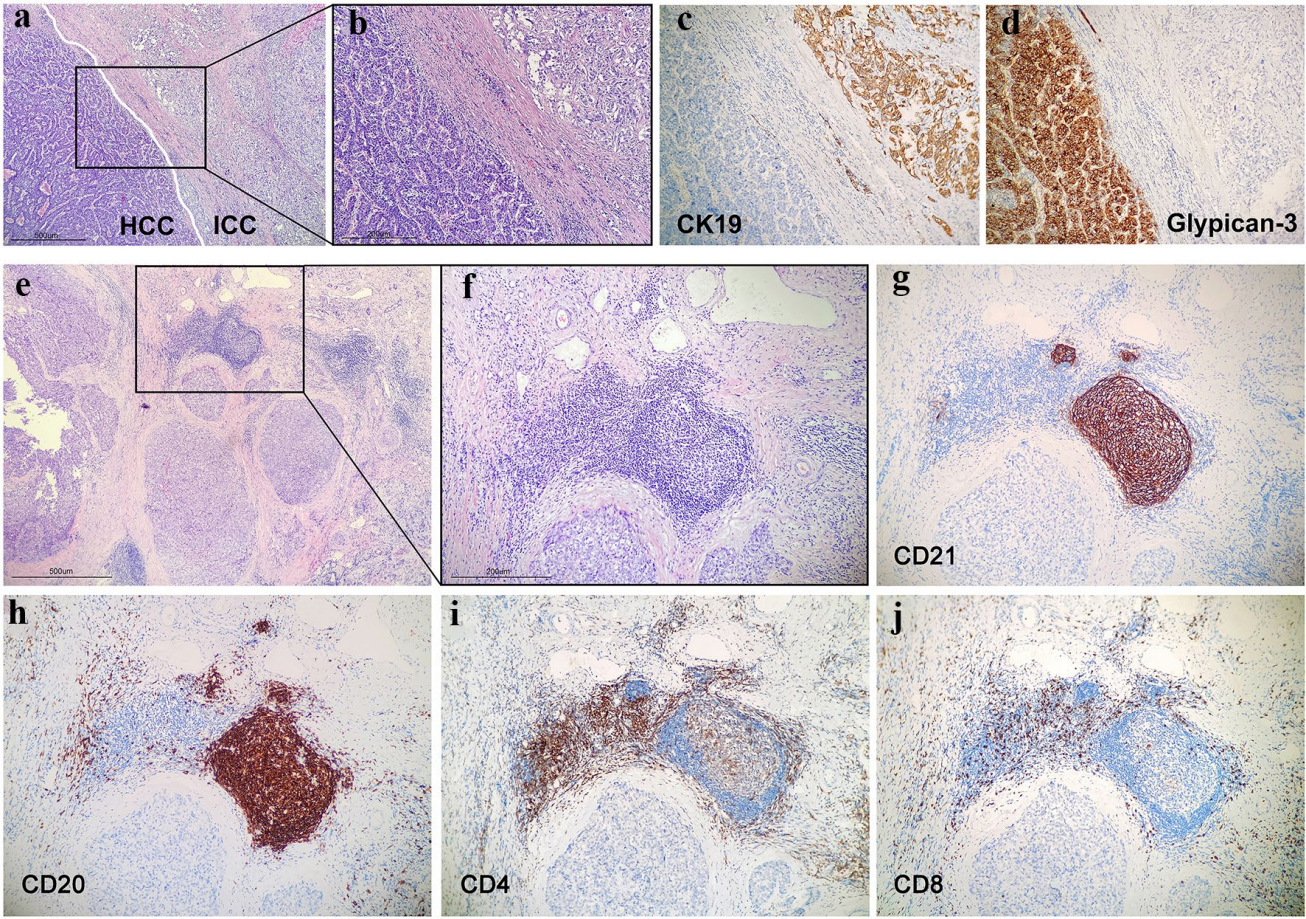
Characteristics of cHCC–CCA and TLS identification. **A**, **B** Representative images of H&E-stained cHCC–CCA tissues consisting of HCC and CCA (40 × and 100 ×). **C** IHC staining showed that CK19 was positively expressed in the CCA component (100 ×). **D** IHC staining showed that Glypican-3 was positively expressed in the HCC component (100 ×). **E**, **F** Representative TLSs of cHCC–CCA tissues were assessed in H&E sections. (40 × and 100 ×). **G** The FDC meshworks in the germinal centre were marked by CD21 (100 ×). **H** The B-cell zone of TLSs was marked by CD20 (100 ×). **I**, **J** The T-cell zone of TLSs was marked by CD4 and CD8 (100 ×). TLSs, tertiary lymphoid structures; HCC, hepatocellular carcinoma; CCA, cholangiocarcinoma; IHC, immunohistochemistry; H&E, haematoxylin–eosin; FDC, follicular dendritic cell

According to the inclusion criteria, 137 patients (111 males) with cHCC–CCA were recruited in the present study. The median age was 56.8 years (36–79), with a median tumor size of 4.75 cm (1–20 cm) (clinicopathological data are summarized in the Supplemental Table). The patients’ median OS and disease‐free survival (DFS) were 22.27 and 17.42 months, respectively. Typical cHCC–CCA consists of two components, HCC and CCA, which intermingle and migrate with each other (Fig. 1A, B) (the other mixing patterns of HCC and CCA components are shown in the Supplementary material Figure S2). The CCA cells were cuboidal with abundant fibrous stroma and arranged in a tubular or cribriform pattern and expressed CK19 (Fig. 1C). HCC cells, characterized by a polygonal shape, were arranged in the form of a beam cord and expressed Glypican-3 (Fig. 1D). The presence and location of TLSs were histologically assessed in H&E sections, which were verified by immunohistochemistry staining. The mature TLS shown in Fig. 1E and F contains a B-cell zone marked by CD20, with a GC marked by CD21 (Fig. 1G, H), and a T-cell zone marked by CD4 or CD8 (Fig. 1I, J).

### The number and distribution of TLSs in cHCC–CCA have different prognostic significances

**Fig. 2:**
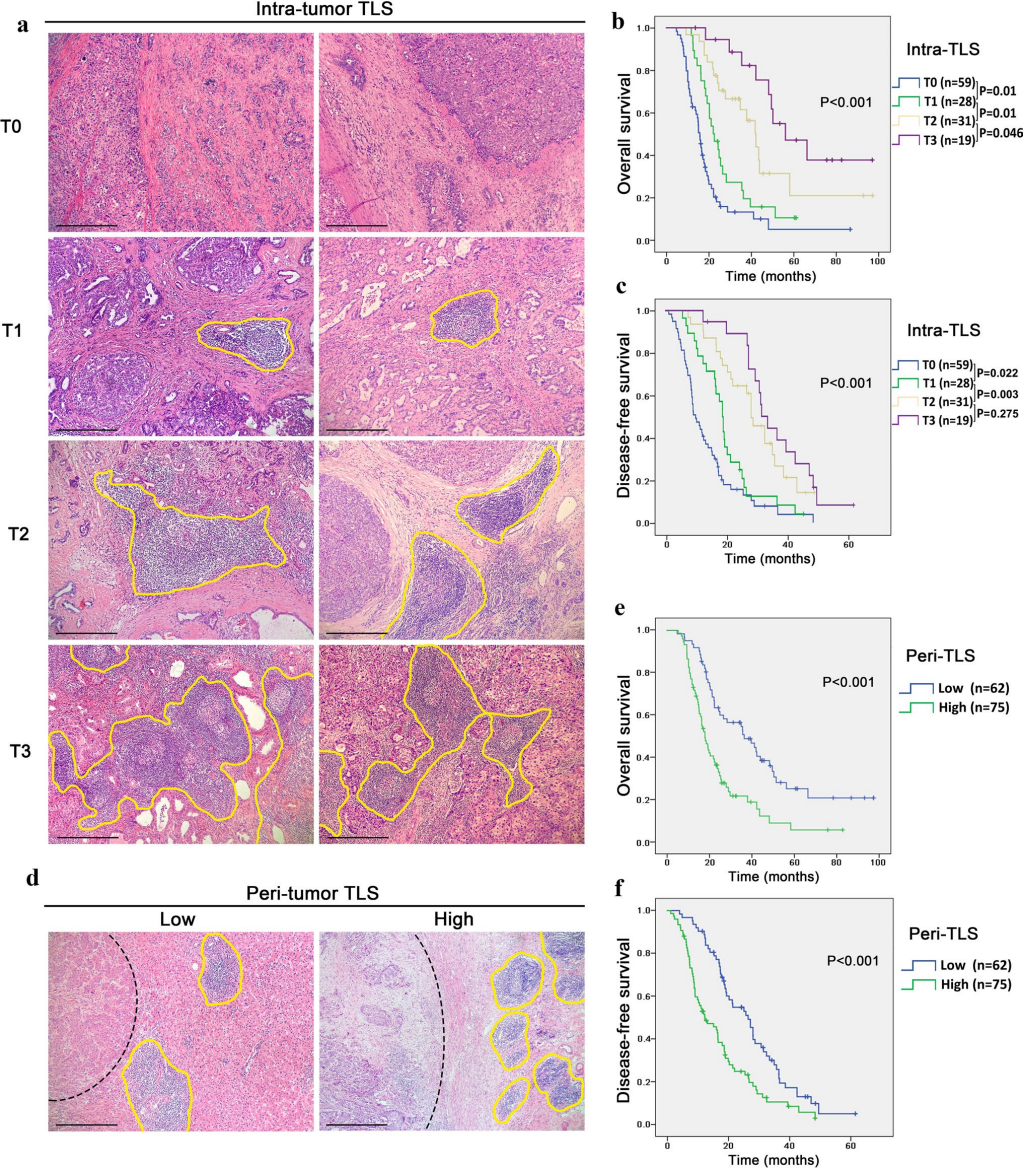
The number and distribution of TLSs in cHCC–CCA have different prognostic significance. **A** Representative images of the intratumoural TLS score system (T0-T3) in H&E-stained sections. TLSs are highlighted by the yellow lines (100 ×). Scale bar: 200 µm. **B**, **C** Kaplan‒Meier analysis was used to compare OS and DFS among patients with T0-T3 intratumoural TLS scores. Significance was tested by the log-rank test. **D** Representative H&E-stained images of peritumoural TLSs in the low-density group and high-density group. Tumor tissue is delineated with a dashed line. TLSs are highlighted by the yellow lines (100 ×). Scale bar: 200 µm. **E**, **F** Kaplan‒Meier analyses were used to compare OS and DFS between the low and high peri-TLS groups. Significance was tested by the log-rank test. TLSs, tertiary lymphoid structures; OS, overall survival; DFS, disease-free survival; H&E, haematoxylin–eosin

According to their distribution, the TLSs of cHCC–CCA were divided into intratumoural TLSs (intra-TLSs) and peritumoural TLSs (peri-TLSs). Tumors with at least one TLS were classified as TLS positive, and tumors without any TLS were classified as TLS negative. The results showed that intra-TLSs were present in 78 (56.9%) patients. Furthermore, according to the number and density of TLSs in the tumor region, TLSs were further divided into four grades: a score of 0 indicated that there were no TLSs in the tumor region (59/137); a score of 1 represented an average tumor region with one or two TLSs per slice (28/137); a score of 2 indicated at least three TLSs in the tumor region, but the density and quantity were lower than those of a score of 3 (31/137); and a score of 3 indicated that a large number of TLSs were distributed in the tumor region and linked to each other (19/137) (Fig. 2A). Survival analysis showed that there was a significant correlation between a high TLS score in tumors and prolonged OS and DFS in cHCC–CCA patients (*p* < 0.001) (Fig. 2B, C). Significant differences in OS were found among each T score group. While there was a significant trend towards longer DFS in patients with higher T scores (T0-T2) (*p* < 0.05), the difference between the DFS of patients with T2 and T3 scores did not reach statistical significance (*p* = 0.275). To investigate the prognostic effect of TLSs in the peritumoural region of cHCC–CCA, peri-TLSs were divided into two groups: the absence of TLS, as well as its limited distribution within a localized area encompassing less than 50% of the region, was both categorized as the Low peri-TLS group (62/137); TLS distribution that spanned over the majority region, exceeding 50%, or extensive TLS encompassing the entire peritumoural region were classified as the High peri-TLS group (75/137) (Fig. 2D). Survival analysis showed that the OS and DFS of patients with a high peri-TLS density decreased significantly (*p* < 0.001) (Fig. 2E, F).

### Maturation of intratumoural TLSs was associated with a good prognosis in cHCC–CCA patients and infiltration of CD8 + T cells in tumors

**Fig. 3:**
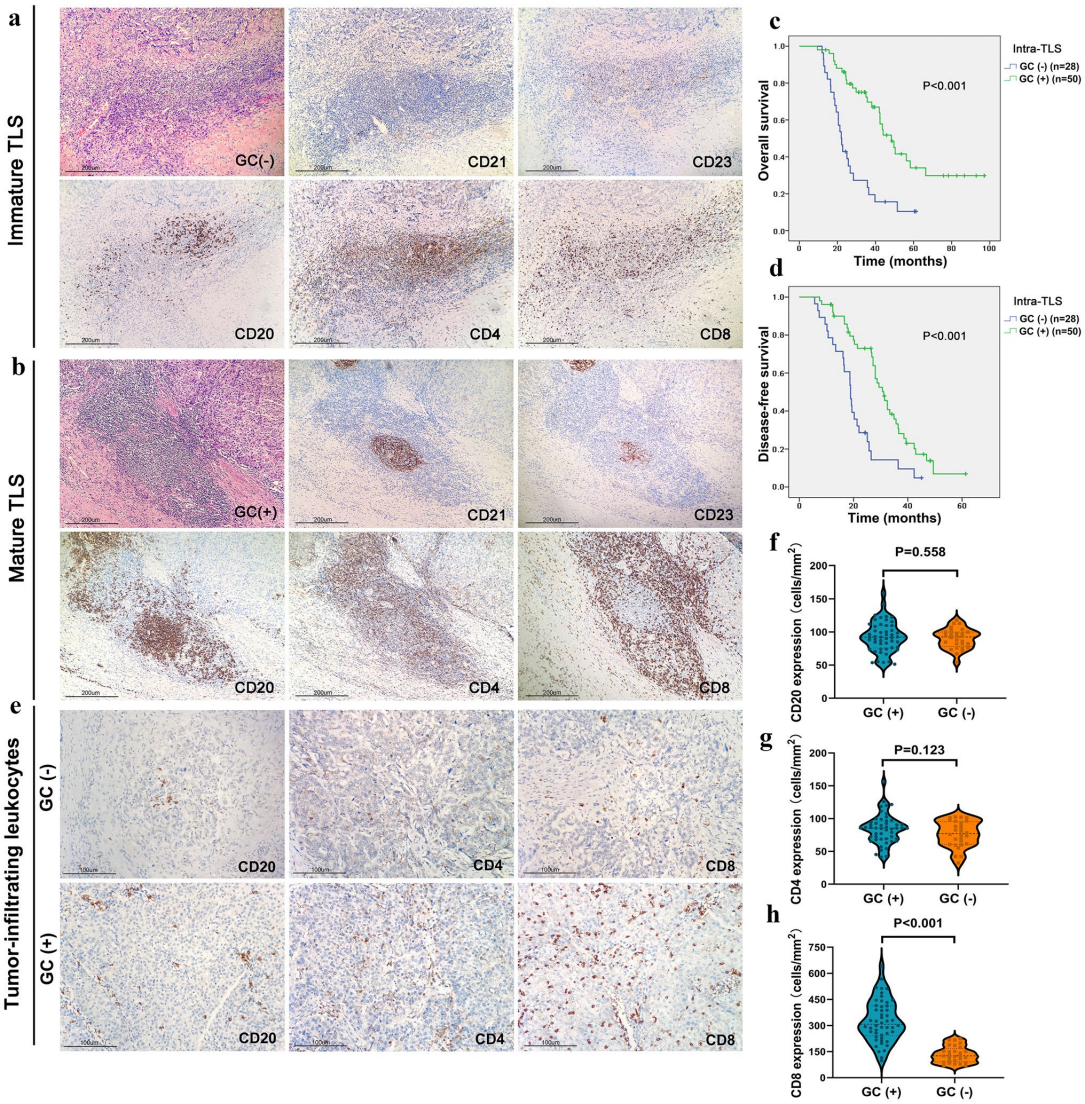
Maturation of TLS is correlated with a favourable prognosis and infiltration of CD8 + T cells in cHCC–CCA tissues. **A**, **B** Representative image of GC (−) and GC ( +) TLSs in cHCC–CCA tissues. The FDC meshworks in GC were labelled by CD21 or CD23. CD20 staining showed the number of B cells. CD4 and CD8 staining showed the number of T cells (100 ×). **C**, **D** Kaplan‒Meier analyses were used for the comparison of OS and DFS between the GC (−) and GC ( +) groups. Significance was tested by the log-rank test. **E** Representative IHC images of tumor-infiltrated CD20 ( +) B cells, CD4 ( +) T cells, and CD8 ( +) T cells in the GC (−) and GC ( +) groups (100 ×). **F–H** Violin plot showing the densities of tumor-infiltrated B and T cells in the GC (-) and GC ( +) groups. GC, germinal centre; TLSs, tertiary lymphoid structures; FDC, follicular dendritic cell; IHC, immunohistochemistry

To clarify the role of TLS maturation in cHCC–CCA, we further analysed the impact of GCs on the prognosis of 78 patients with TLSs. The results showed that the presence of characteristic GC morphology was observed in 50/78 (64.1%) tumors. The number of cases with lymphocyte aggregation or primary lymphoid follicles, considered as cases without GCs, was 28/78 (35.9%) (Fig. 3A, B). Survival analysis showed that the OS and DFS of patients with GCs were longer than those of patients without GCs (*p* < 0.001) (Fig. 3C, D). These results indicate that the maturation of TLSs in cHCC–CCA tumors is significantly related to the favorable prognosis of patients. We further analysed the relationship between lymphocytic infiltration in tumors and the maturation of GCs. The results showed that there were more CD8 + T cells infiltrating the tumor tissues of patients with mature TLSs (*p* < 0.05) (Fig. 3E). At the same time, there was no significant difference between the infiltration of CD20 + B cells and CD4 + T cells (Fig. 3F-H). These results suggested that cytotoxic T-cell infiltration into the tumors may play an essential role in the difference between the two groups.

### Immune grade combined with the cHCC–CCA peri-TLS and intra-TLS scores has prognostic significance

**Fig. 4:**
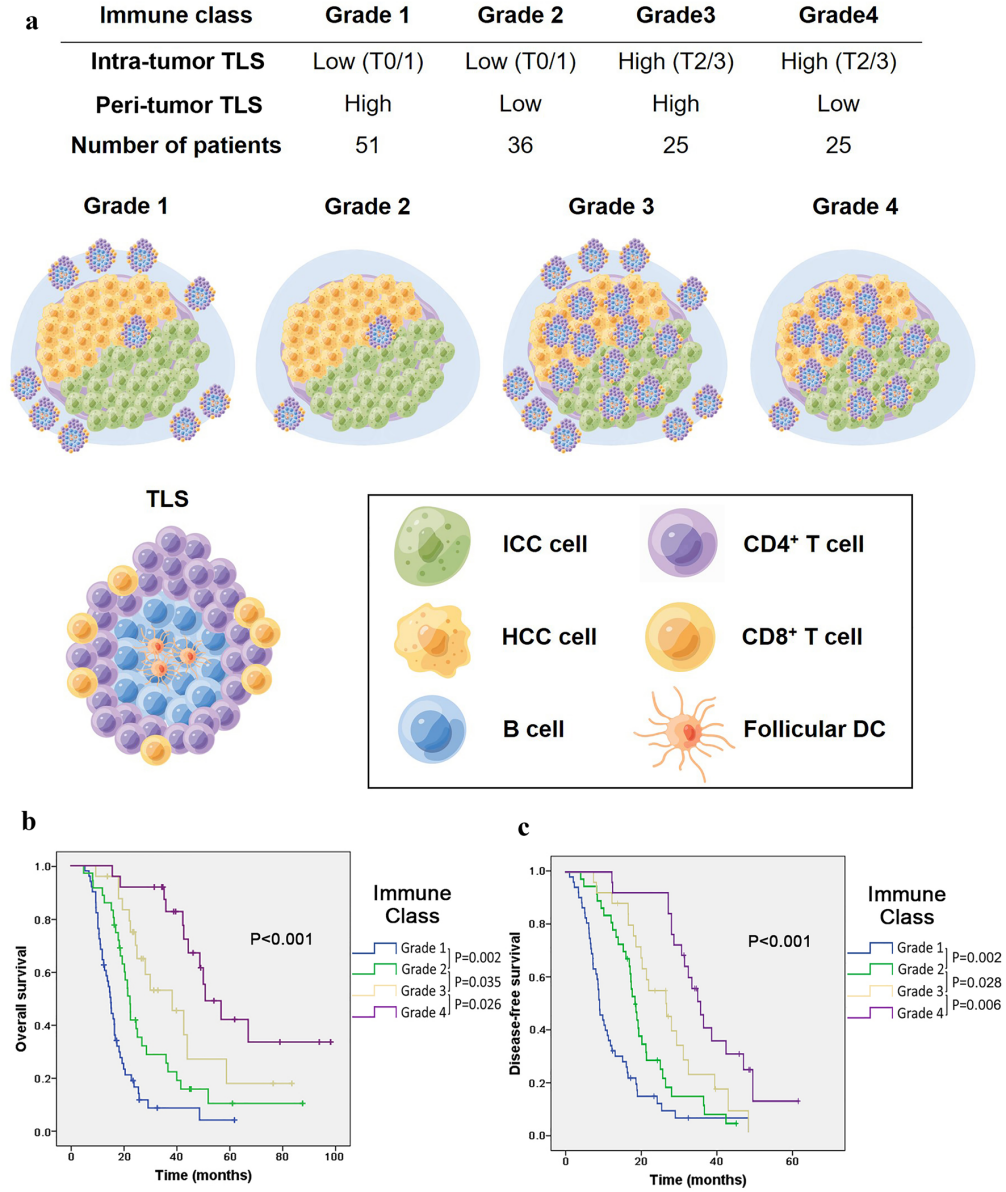
Immune grade combined with the cHCC–CCA peri-TLS and intra-TLS scores has prognostic significance. **A** The model schematic shows that tumors are stratified into four immune grades according to the intra-TLS and peri-TLS. **B**, **C** Kaplan‒Meier analyses were used to compare OS and DFS among the four immune grades. Significance was tested by the log-rank test. OS, overall survival; DFS, disease-free survival

Given the varied significance of the distribution of TLSs in cHCC–CCA tissues, we further divided the patients into four immune levels according to the status of different immune environments by combining peri-TLSs with intra-TLSs. The results showed that 51 (37.2%) cases with TLSs mainly located in the peritumoural region were classified as grade 1; 36 (26.3%) cases with low TLS density in the intratumoural and peritumoural regions were classified as grade 2; 25 (18.2%) cases with relatively high TLS density in both the intratumoural and peritumoural regions were classified as grade 3; and 25 (18.2%) cases with high TLS density mainly located in the intratumoural region were classified as grade 4 (Fig. 4A). Kaplan‒Meier survival analysis demonstrated that an increasing grade score was associated with an incrementally improved OS (median OS: grade 1, 14.8 months vs. grade 2, 21.4 months vs. grade 3, 26.5 months vs. grade 4, 45.8 months; Fig. 4B). Consistently, the high immune grade of cHCC–CCA patients was associated with a prolonged DFS (median DFS: grade 1, 8.9 months vs. grade 2, 18.1 months vs. grade 3, 22.0 months vs. grade 4, 32.4 months; Fig. 4C), and the differences in OS and DFS between each immune grade were statistically significant (*p* < 0.05). Univariate and multivariate Cox analyses were performed to analyse the effects of clinicopathological characteristics, CXCL12, and TLS grade on the OS of cHCC–CCA patients. Multivariable Cox analysis identified tumor size (HR = 1.61; 95% CI: 1.059–2.447; *p* = 0.026), histological grade (HR = 3.906; 95% CI: 2.334–6.535; *p* < 0.001), CA19-9 level (HR = 1.64; 95% CI: 1.072–2.509; *p* = 0.023) and TLS grade (HR = 0.554; 95% CI: 0.451–0.681; *p* < 0.001) as independent prognostic factors for OS (Table 1).

**Table 1:** Univariate and multivariate analysis of prognostic factors for overall survival in cHCC–CCA

Characteristics	Univariate analysis				Multivariate analysis		
	HR	95% CI	*p*		HR	95% CI	*p*
Age	1.016	0.663–1.556	0.944				
Sex	0.990	0.593–1.652	0.968				
Tumor size (≥ 5 cm)	1.675	1.120–2.505	0.012*		1.610	1.059–2.447	0.026*
Satellite nodule	2.691	1.792–4.040	0.000*				
Histological grade	4.834	3.019–7.741	0.000*		3.906	2.334–6.535	0.000*
CCA percentage (≥ 50%)	1.567	1.021–2.405	0.040*				
Cirrhosis	1.025	0.692–1.519	0.901				
HBV	0.913	0.597–1.397	0.676				
Macrovascular invasion	1.593	1.038–2.444	0.033*				
Microvascular invasion	1.303	0.855–1.985	0.219				
Lymph node metastasis	1.676	1.097–2.562	0.017*				
CA19-9 (≥ 25 u/ml)	1.566	1.028–2.386	0.021*		1.640	1.072–2.509	0.023*
AFP20 (≥ 20 ng/ml)	1.037	0.700–1.536	0.856				
CXCL12	0.631	0.425–0.936	0.022*				
TLS grade	0.489	0.398–0.600	0.000*		0.554	0.451–0.681	0.000*

### Potential predicting value of TLS for immunotherapy response in cHCC–CCA cases

In view of the role of TLSs in evaluating tumor immune status, we further explored their role in the cHCC–CCA immunotherapy response, which had not been reported. Although cHCC–CCA is a rare type of tumor and it is difficult to confirm the diagnosis by needle biopsy specimens before treatment, we were fortunate to obtain two recently confirmed patients with cHCC–CCA at high risk of recurrence, following treatment with carrelizumab and apatinib, which are recommended as first-line standard regimens for advanced HCC in the guidelines of the Chinese Society of Clinical Oncology according to the RESCUE study. We used these two case reports to preliminarily evaluate the response of cHCC–CCA to immunotherapy.

**Fig. 5:**
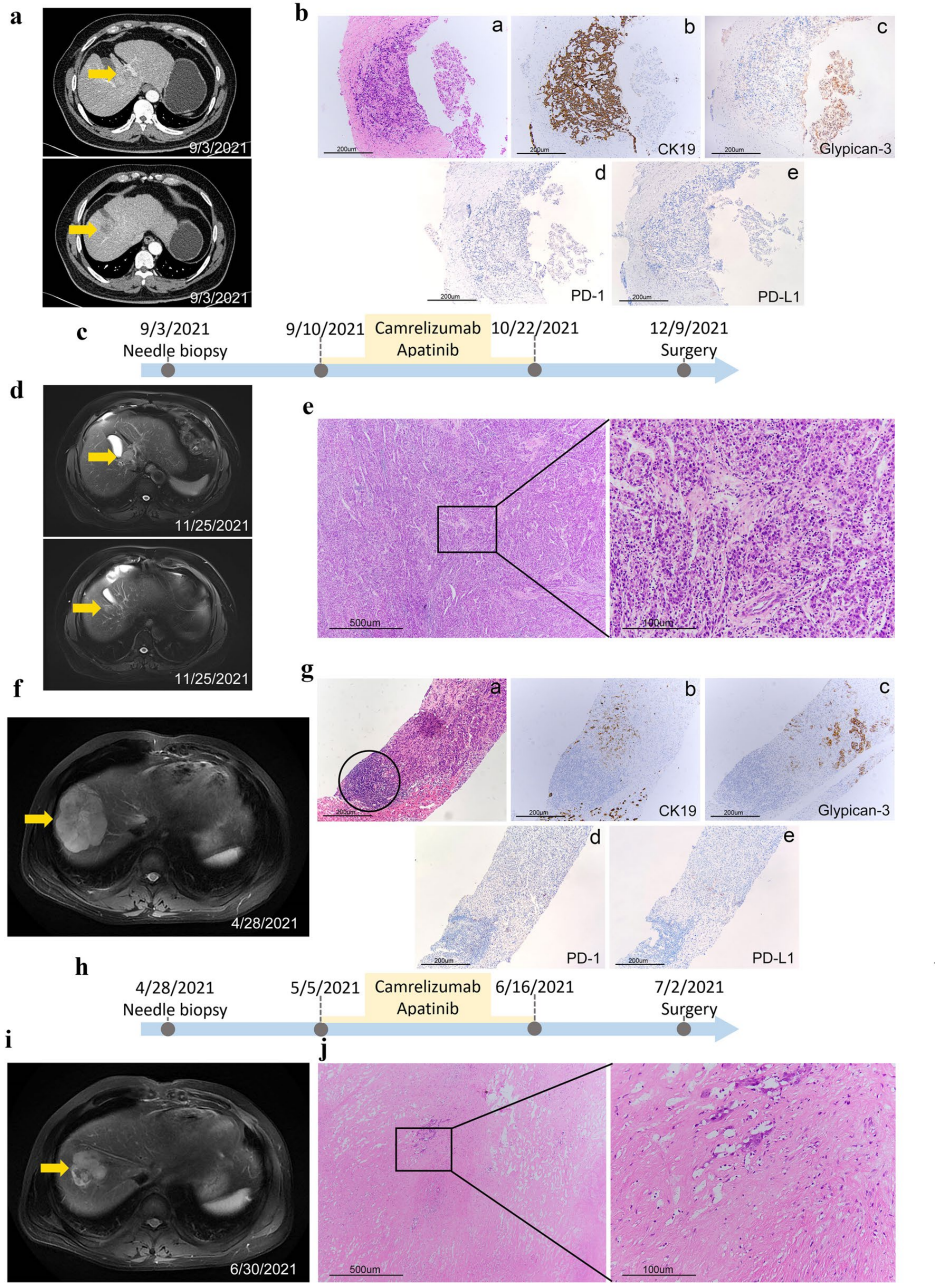
Prediction of cHCC–CCA immunotherapy response by TLSs. **A** Pretherapy abdominal enhanced CT of Case 1 revealed two masses in the left inner lobe and right upper lobe of the liver (yellow arrow). **B** H&E, CK19, glypican-3, PD-1, and PD-L1 staining of tumor biopsy tissue (100 ×). **C** Timeline of the treatment process of Case 1. **D** Posttherapy MRI showed that the tumor volume did not change significantly (yellow arrow). **E** Representative IHC staining images of postoperative specimens (40 × and 200 ×). **F** Pretherapy MRI of Case 2 revealed a mass in the S8 segment of the liver (yellow arrow). **G** H&E, CK19, Glypican-3, PD-1, and PD-L1 staining of tumor biopsy tissue (100 ×). TLSs could be seen in tumor tissue (a, black circle). **H** Timeline of the treatment process of Case 2. **I** Posttherapy MRI showed that the tumor volume was significantly reduced (yellow arrow). **J** Representative IHC staining images of postoperative specimens (40 × and 200 ×). CT, computed tomography; H&E, haematoxylin–eosin; MRI, magnetic resonance imaging; PD-1, programmed death-1; PD-L1, programmed death ligand 1

Case 1 was a patient in their 50s admitted in September 2021, and abdominal enhanced CT showed two uneven soft tissue masses in the left inner lobe of the liver (2.2 × 1.5 cm) and right upper lobe (2 × 1.6 cm) (Fig. 5A). On 9/1/2021, a needle biopsy was performed, and IHC staining showed that the HCC components Glypican-3, Arginase-1, Hepatocyte, and Glutamine synthetase (GS) were positive, while the CCA components CK19 and CK7 were positive, and Ki67 showed approximately 40% cell-positive staining. This patient was diagnosed with cHCC–CCA, and no TLSs were present in the tumor tissues (Fig. 5Ba-c). In addition, PD-1 and PD-L1 IHC staining showed negative expression (Fig. 5Bd, e). After receiving carrelizumab and apatinib preoperative neoadjuvant therapy on 9/10/2021 for four cycles (Fig. 5C), the MRI result on 11/25/2021 revealed that the volume of the tumors did not show significant changes (2.4 × 2.1 × 1.8 cm and 2 × 1.8 × 1.6 cm) (Fig. 5D). The assessment of the tumor via Recist1.1 by videography indicated stable disease (SD). The patient underwent 4–5 segment resection of the liver on December 9, and postoperative pathological examination showed that the tumor was non-necrotic, and there was a small amount of lymphocyte infiltration in the tumor stroma (Fig. 5E).

Case 2 was a patient in their 40s who was admitted in April 2021, and an abdominal MRI demonstrated a mass with heterogeneous enhancement (7.6 × 6.3 × 5.8 cm) (Fig. 5F). On April 29, 2021, a needle biopsy was performed, and the IHC results suggested the diagnosis of cHCC–CCA (Fig. 5 Ga-c). Similarly, the expression of PD-1 and PD-L1 in tumor tissue was negative, except for several positively stained lymphocytes (Fig. 5Gd, e). TLSs could be seen in the tumor tissue (Fig. 5G, black circle). The patient also received carrelizumab and apatinib preoperative neoadjuvant therapy on May 5, 2021 for four cycles (Fig. 5H). MRI on June 30 showed that the tumor volume was significantly reduced to 5.5 × 4.4 × 3.7 cm (Fig. 5I). The assessment of the tumor via Recist1.1 by videography indicated a partial response (PR). The patient underwent S8 segment resection of the liver on July 2, and postoperative pathological examination showed massive necrosis of the tumor (necrosis rate approximately 80%) (Fig. 5J).

**Fig. 6:**
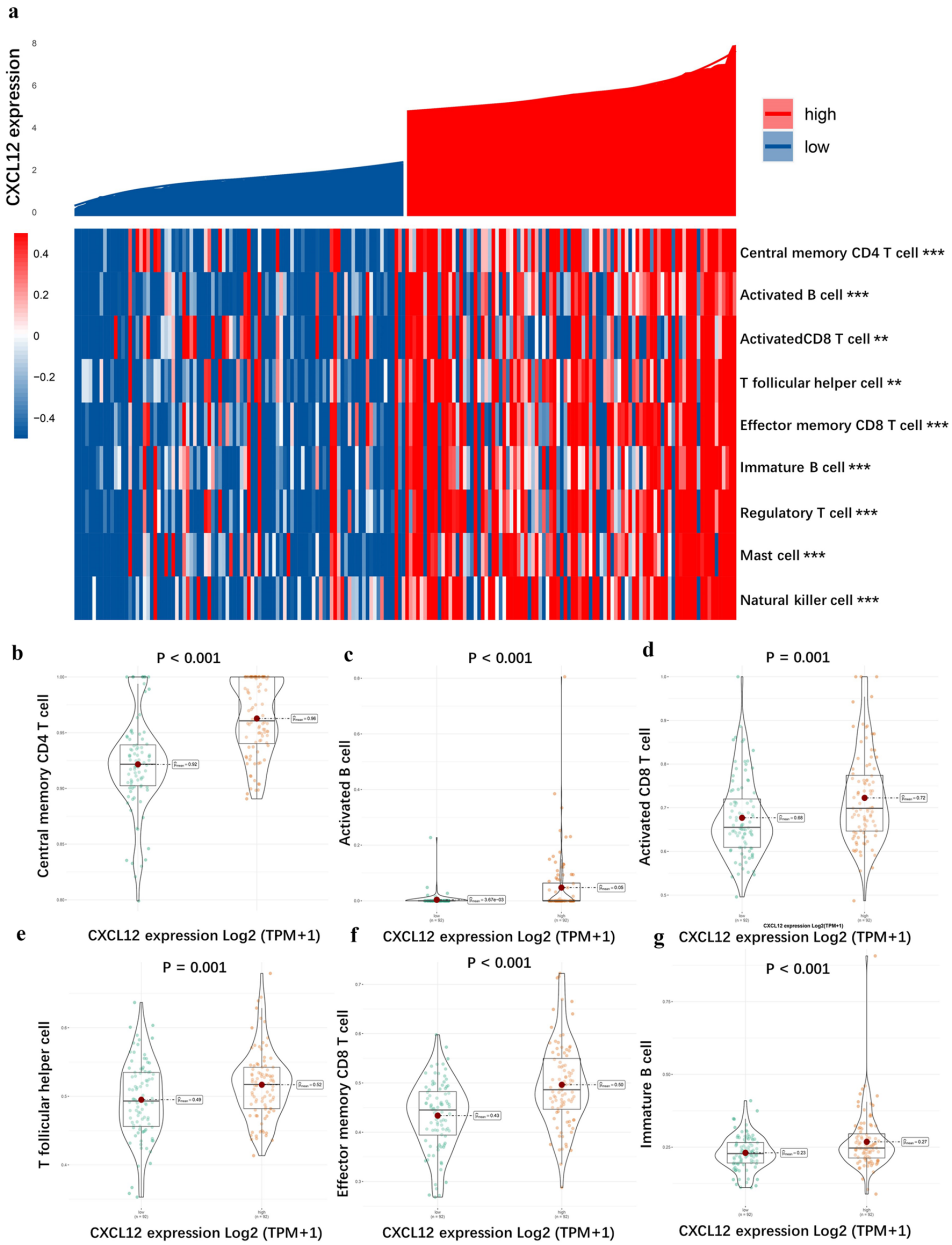
Association of CXCL12 expression and immune cells infiltration in LIHC of TCGA cohort. **A** Immune cell infiltration analysis in 373 liver hepatocellular carcinoma (LIHC) samples from the TCGA cohort ordered by increasing CXCL12 expression. **B**–**G** Association of immune population signatures and CXCL12 expression in LIHC

The tumor of case 2 was significantly reduced, and most of the tumor tissue became necrotic after immunotherapy treatment compared with case 1. Our results indicated that even if PD-1 and PD-L1 were not expressed, TLSs in tumor tissue might be used as a potential indicator for predicting the immunotherapeutic response. However, the number of cases reported in the present study was limited, and a prospective study will be performed in future studies to verify the clinical implications of TLSs in cHCC–CCA.

### Correlation between CXCL12 and immune cell infiltration

We performed an immune infiltration analysis in liver hepatocellular carcinoma from the Cancer Genome Atlas (TCGA) and Gene Expression Omnibus (GEO) database as an example. The correlation between CXCL12 and immune cell infiltration in 373 liver hepatocellular carcinoma (LIHC) samples from TCGA was examined using TISIDB. ( http://cis.hku.hk/TISIDB). The infiltrating immune cell subtypes, such as central memory CD4 T cell, activated B cell, activated CD8 T cell, T follicular helper cell, effector memory CD8 T cell, immature B cell, regulatory T cell, mast cell, and natural killer cell, were all significantly associated with CXCL12 expression as indicated in Spearman's correlation analysis (Fig. 6). Similarly, CXCL12 expression was found to be correlated with abundance of immune cells in the GSE76427 cohort (Supplementary Figure S1). The recruitment and increased infiltration of immune cells in tumor tissue may suggest the presence of TLS. In conclusion, these results indicate CXCL12 expression closely related to a high aggregation of tumor infiltrating immune cells in hepatocellular carcinoma tissues and that high CXCL12 expression cases have an immune microenvironment adapted to TLS formation.

### CXCL12 expression is significantly correlated with TLS formation in cHCC–CCA tissue

**Fig. 7:**
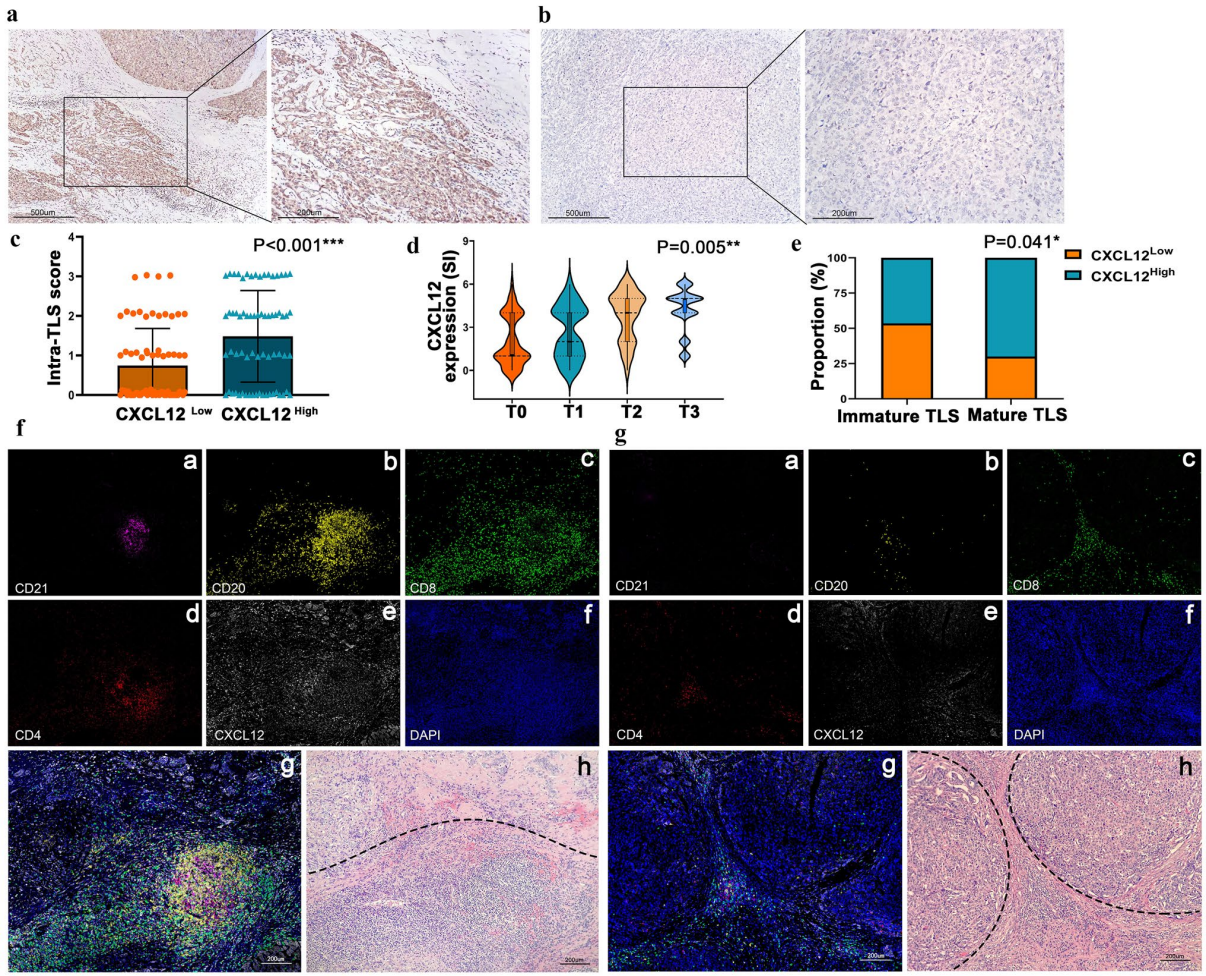
CXCL12 expression is correlated with TLS formation in cHCC–CCA tissue. **A**, **B** Representative IHC staining images of high and low CXCL12 expression in cHCC–CCA tissues (100 × and 200 ×). **C** Scatter plot showing the intra-TLS score of the CXCL12 low and high groups in cHCC–CCA patients. **D** Violin plot showing that a higher T score was associated with a higher CXCL12 staining index (SI). **E** Stacked column chart showing that the mature TLS group exhibited a higher rate of CXCL12 expression. **F** Representative fluorescent multiplex immunohistochemistry images show the positivity of CD21 (a, pink), CD20 (b, yellow), CD8 (c, green), and CD4 (d, red) in intratumoural TLSs and CXCL12 (e, white) expression. The nuclei were labelled with DAPI (f, blue). Merged image (g) and corresponding H&E-stained image (h). Tumor tissue is delineated with a dashed line. Scale bar: 200 µm. **G** Representative multiplex immunostaining images of lymphocyte aggregation. Tumor tissue is delineated with a dashed line. Scale bar: 200 µm. IHC, immunohistochemistry; TLSs, tertiary lymphoid structures; H&E, haematoxylin–eosin

In view of the role of CXCL12 in lymphocyte recruitment and GC formation in normal lymphoid tissues, we preliminarily explored the relationship between its expression in cHCC–CCA tumor tissue and the formation of TLSs. As depicted in Fig. 7A-C, correlation analysis revealed that the expression of CXCL12 in cHCC–CCA tissue was significantly correlated with the intratumoural TLS score (*p* < 0.001). A higher T score was associated with a higher CXCL12 staining index (SI) (*p* = 0.005, Fig. 7D). Moreover, the results indicated that 35/50 (70%) cases in the mature TLS group exhibited high CXCL12 expression. In the immature TLS group, 13/28 (46.43%) cases displayed high CXCL12 expression, and the difference was statistically significant (*p* = 0.041) (Fig. 7E). The relationships between TLSs and CXCL12 expression and location were further verified by fluorescent multiplex immunohistochemistry staining, as shown in Fig. 7F and G. CXCL12 was highly expressed in cancer tissue in the TLS high-grade group. In contrast, CXCL12 expression was relatively low in cancer tissue in cases with only a few lymphocyte aggregates.

## Discussion

cHCC–CCA, as a rare tumor, is challenging to accurately assess in terms of the clinical outcomes and prognostic risk factors in patients. More importantly, its high heterogeneity contributes to unsatisfactory treatment outcomes. Therefore, it is meaningful to explore prognostic and therapeutic markers to help physicians accurately stratify cHCC–CCA patients [20]. In recent years, despite the gradual improvement in the prognosis of cHCC–CCA patients, there are still no effective therapeutic treatments, thereby leading to a frustratingly low survival rate [3]. Recent studies have shown that TLSs provide a crucial local microenvironment for cellular and humoral immune responses against tumor cells and are considered a marker of favourable clinical outcomes for various malignancies. However, their presence is highly variable between cancer types and patients [6]. This study is the first to clarify the role and significance of TLSs in cHCC–CCA with a relatively large patient cohort. Our results showed that intra-TLSs were present in 78 (56.9%) patients, and survival analysis demonstrated that there was a significant correlation between a high TLS score in tumors and prolonged OS and DFS in cHCC–CCA patients (*p* < 0.001). In contrast, the survival time of patients with a high peri-TLS density in adjacent tissue decreased significantly (*p* < 0.001). Recent studies demonstrated that TLSs can be used to predict the prognosis of patients with PLC, and their distribution has different predictive significance. A previous study in CCA suggested that intra-TLSs positively correlated with a favourable prognosis, whereas a high peri-TLS signified a worse survival. However, it also reported that high pTLS density represents a promising prognostic biomarker for HCC [9, 10]. Therefore, there were still discrepancies in the significance of TLS around tumors in different types of PLC, which may be attributed to the immune escape ability of different tumor types and the inflammatory status of adjacent liver tissue. As a rare primary hepatic tumor, the specific role of TLS in cHCC–CCA remains unclear. Our research findings indicated that the prognostic significance of TLS in cHCC–CCA was consistent with that of CCA, but the specific role and underlying mechanisms require further investigation. In addition, our results indicated that the maturation of TLSs in cHCC–CCA tumors was significantly related to more cytotoxic CD8 + T cells infiltrating tumor tissues, which target tumor cells and play essential roles in effective antitumour immunity, thereby improving the prognosis of patients. Similarly, some studies reported that the presence of TLSs in tumors was accompanied by an increase in immune infiltration, and intratumoural CD8 + T-cell infiltrates were correlated with a superior prognosis [21].

Furthermore, relevant studies have emphasized the significance of TLS phenotypic maturity and spatial distribution [22]. In view of the varied significance of the distribution of TLSs in cHCC–CCA tissues, the patients were further divided into four immune grades according to the status of different immune environments by combining peri-TLSs and intra-TLSs. Kaplan‒Meier survival analysis demonstrated that an increase in the grade score was associated with incrementally improved OS and DFS, and there was a significant difference between each immune grade (*p* < 0.05). The varied cell composition of TLSs within and around the tumor may determine their pro- or antitumour activities, which is consistent with the results of previous studies on ICC [10]. In the present study, we speculated that grade 1 was characterized by the lowest antitumour response, indicating a solid immune exclusion ability of the tumor, resulting in the worst prognosis. Grade 2 represented that the tumor and its adjacent areas had a lower immune level. Compared with grade 1 TLSs, peritumour TLSs exhibited a lower degree of inflammation, which was not conducive to tumor growth and migration. Grade 3 tumors showed a high level of antitumour immunity, but the antitumour immunity effect was also sabotaged by the surrounding inflammatory state. Patients with grade 4 tumors exhibited more intratumoural TLSs and fewer peritumoural TLSs, showing an active and effective antitumour immune response, indicating an optimal prognosis. Multivariable Cox analysis indicated that tumor size, differentiation grade, CA19-9 level, and TLS grade were independent prognostic factors for the OS of cHCC–CCA.

Despite the effectiveness of immune checkpoint inhibitors (ICIs) in PLC, the overall objective response rate remains less than 40%, resulting in the inability of some patients to benefit from them [23]. Regarding the response or drug resistance of PLC immunotherapy, there are no universally recognized and widely used predictive indicators in the current clinical guidelines. Response prediction based on the features of immune characteristics is considered a critical step amid various approaches to improve the success rate of ICI treatment [24], especially for PLC tissues with relatively low positive rates of PD1 or PD-L1 expression [25]. Recent studies have successfully demonstrated the correlation between TLSs and immunotherapy in melanoma and sarcoma, indicating that the formation of TLSs is associated with a favourable response to ICI treatment [11, 12]. A case report study indicated that the tumour immune microenvironment has the potential to offer insights into the combined immunotherapy response for HCC [26]. However, there are no studies related to cHCC–CCA at present. In this study, we reported the treatment response of two patients with cHCC–CCA to carrelizumab combined with apatinib. The presence of TLSs was observed in the biopsy tissue of one patient, with the tumor significantly reduced and most of the tumor tissue necrotic after treatment compared with the other patient. This exciting result indicated that TLSs might serve as a potential indicator for predicting the immunotherapeutic response, even in tumors with negative PD-1/PD-L1 expression. Despite limitations in diagnosis and TLS evaluation, biopsy tissues can still be used to predict the response to immunotherapy if the immune status can be assessed. In addition, even after the diagnosis and TLS assessment of the postoperative specimens, immunotherapy for patients with cHCC–CCA who could derive a benefit will also prominently improve their prognosis by preventing recurrence and distant metastasis. The prospective study will be performed in future research to verify the predictive effect of TLS on immunotherapy implications in the recurrence prevention of cHCC–CCA. Although the number of cases reported in the present retrospective study is limited, we hope to shed new light on immunotherapy for patients with cHCC–CCA. Our findings will be further validated through future research encompassing a larger sample size and a prospective clinical trial of cHCC–CCA.

The CXCL family holds a pivotal position in TLS formation [6], while CXCL12 is particularly important in the formation of lymphoid follicles. Previous studies reported that CXCL12 is responsible for B-cell lymphopoiesis and plays crucial roles in the migration and repositioning of B cells within the GC [16, 27]. Additionally, the aggregation of B cells is fundamental in the initial stages of TLS formation [6]. CXCL12 was first identified as a signal from the bone marrow microenvironment to regulate haematopoiesis. In the liver, CXCL12 is produced by bile duct epithelial cells, hepatic stellate cells, sinusoidal endothelial cells, and malignant cells [15]. Because CXCL12 and CXCR4/CXCR7 play important roles in liver homeostasis and regeneration, their expression has complex interactions in the pathogenesis of liver diseases [28]. Several studies have shown that the CXCL12 pathway is involved in the progression of chronic liver disease and liver fibrosis [29]. Some studies have shown that CXCR4 is upregulated in human HCC cells, and the CXCL12–CXCR4 axis promotes tumor migration and angiogenesis in HCC [30]. However, some studies have shown that patients with relatively high CXCL12 expression in tumors have prolonged OS. High CXCL12 expression in tumors may desensitize tumor cells expressing CXCR4 receptors, thus preventing tumor proliferation [17, 31]. Previous studies have demonstrated that CXCL12 can be produced by tumor cells of HCC and ICC [30–33]. However, these studies primarily concentrated on the impact of CXCL12 on tumor progression. While our study aims to explore the effects of tumor-derived CXCL12 on the tumor immune microenvironment and TLS formation. Given the significant role of CXCL12 in lymphoid follicles, we analysed the association between CXCL12 expression and immune infiltration in HCC tissues using the TCGA and GEO databases, and found that high CXCL12 expression in tumor tissues was significantly correlated with a high-level immune cell infiltration. We speculate that the infiltration of immune cells into the interior of tumors that have escaped immune surveillance requires the secretion of recruitment factors by tumor cells. Our findings demonstrated that the expression of CXCL12 in cHCC–CCA tumor cells was positively correlated with the intratumoural TLS score and TLS maturation. The results suggest that CXCL12 expression may promote the formation and maturation of TLSs, thereby promoting a favourable prognosis in patients. We speculate that the overexpression of CXCL12 might play a significant role in B-cell migration, leading to the aggregation of TLSs and the formation of GCs. However, it is important to note that the formation of TLSs is a highly intricate process, involving a complex network of cells, molecules, and their interactions, which makes fully elucidating its mechanisms challenging. Despite the limitations of current research, our findings nonetheless provide valuable insights into the formation of TLS in cHCC–CCA, offering possible inspiration for future research.

In summary, we demonstrate that a high intra-TLS score was related to prolonged OS and DFS, whereas a high peri-TLS score in adjacent tissue indicated a worse prognosis. Our findings also revealed that mature TLSs were related to favourable outcomes and showed more cytotoxic CD8 + T cells infiltrating tumor tissues. A novel immune classification combining the spatial distribution and density of TLSs showed significant prognostic value. In addition, our reported cases suggested a potential value of TLS in predicting immunotherapy response in cHCC–CCA patients, but more validations are needed. Moreover, we preliminarily demonstrated the positive correlation between CXCL12 expression and TLS presence in cHCC–CCA, and further research should clarify the mechanism and function. Overall, the present study may provide new insights into the clinical decisions and stratification of cHCC–CCA patients.

### Supplementary Information

Below is the link to the electronic supplementary material.

Supplementary file1 (DOCX 3558 KB)

## Data Availability

The original contributions presented in the study are included in the article/supplementary material, and further inquiries can be directed to the corresponding authors.
