# Supplementary material for: Tertiary lymphoid structures as a potential prognostic biomarker for combined hepatocellular–cholangiocarcinoma
Source: Hepatol Int. 2024 May 20;18(4):1310–25. doi: 10.1007/s12072-024-10694-2 (PMC11297834; doi:10.1007/s12072-024-10694-2)
Supplement: Supplementary file 1 — Supplementary file1 (DOCX 3558 KB) [file 12072_2024_10694_MOESM1_ESM.docx]

**Supplement Table Clinicopathological data of the enrolled cases.**

| **Characteristics** | **N** | **Proportion** |
| --- | --- | --- |
| Sex |  |  |
| Male | 111 | 81.00% |
| Female | 26 | 19.00% |
| Age (years) |  |  |
| < 60 | 88 | 64.23% |
| ≥ 60 | 49 | 35.77% |
| Cirrhosis |  |  |
| Negative | 70 | 51.09% |
| Positive | 67 | 48.91% |
| HBV |  |  |
| Absent | 42 | 30.66% |
| Present | 95 | 69.34% |
| Tumor size |  |  |
| < 5cm | 66 | 48.18% |
| ≥ 5cm | 71 | 51.82% |
| Histological grade |  |  |
| High/Median | 80 | 58.39% |
| Low | 57 | 41.61% |
| Satellite nodule |  |  |
| Absent | 79 | 57.66% |
| Present | 58 | 42.34% |
| Macrovascular invasion |  |  |
| Absent | 104 | 75.91% |
| Present | 33 | 24.09% |
| Microvascular invasion |  |  |
| Absent | 97 | 70.80% |
| Present | 40 | 29.20% |
| Lymph node metastasis |  |  |
| Absent | 102 | 74.45% |
| Present | 35 | 25.55% |
| AFP |  |  |
| < 20 ng/ml | 69 | 50.37% |
| ≥ 20 ng/ml | 68 | 49.63% |
| CA19-9 (> 25u/ml) |  |  |
| < 25 u/ml | 54 | 39.42% |
| ≥ 25 u/ml | 83 | 60.58% |
| CXCL12 |  |  |
| Low | 69 | 50.37% |
| High | 68 | 49.63% |
| CCA percentage (≥50%) |  |  |
| < 50% | 50 | 36.50% |
| ≥50% | 87 | 63.50% |
| Intra-TLS |  |  |
| T0 | 59 | 43.06% |
| T1 | 28 | 20.44% |
| T2 | 31 | 22.63% |
| T3 | 19 | 13.87% |
| Peri-TLS |  |  |
| Low | 62 | 45.26% |
| High | 75 | 54.74% |

**
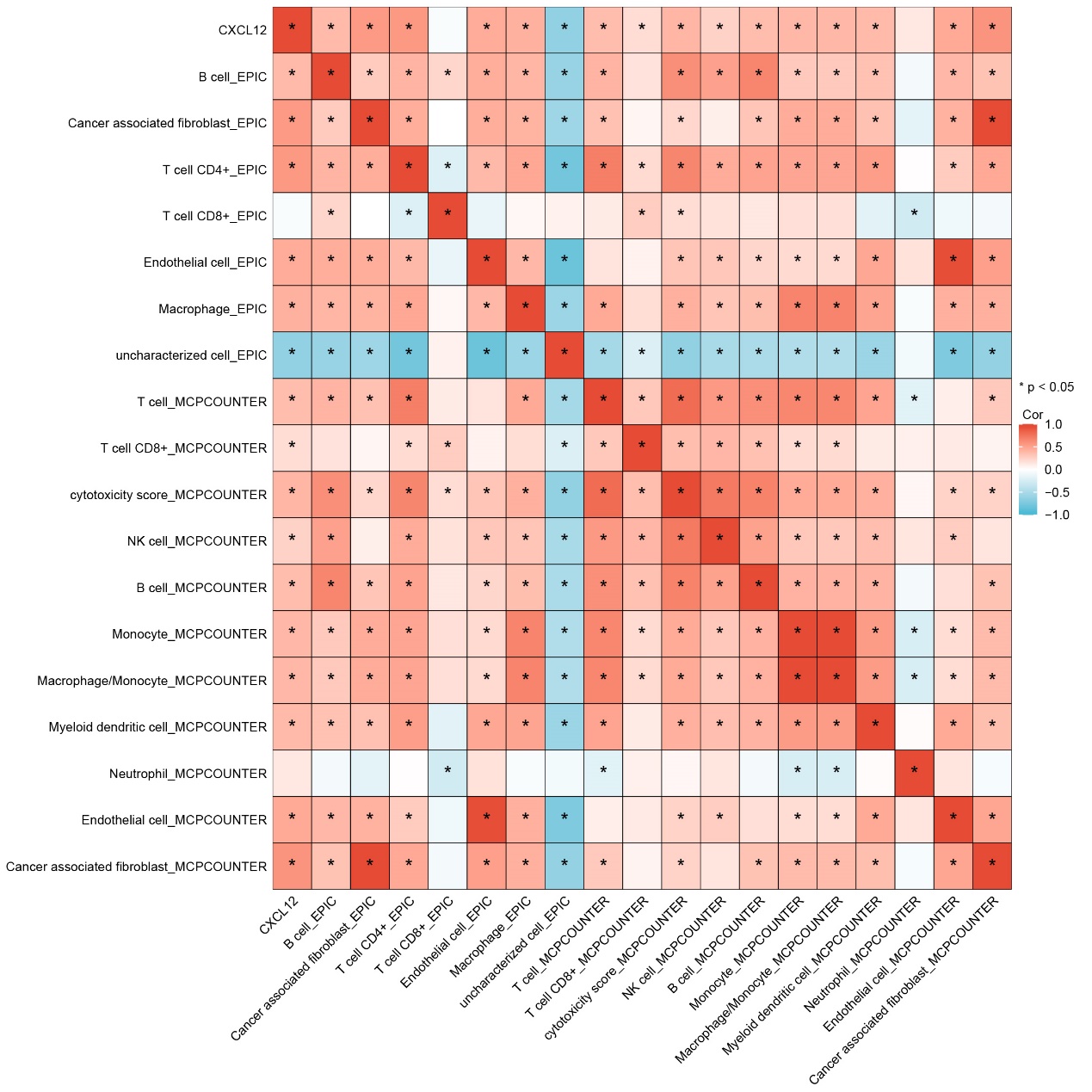
**

**Figure S1. Association of CXCL12 expression and immune cells infiltration in 115 HCC patients’ cohort from Gene Expression Omnibus (GSE76427).**


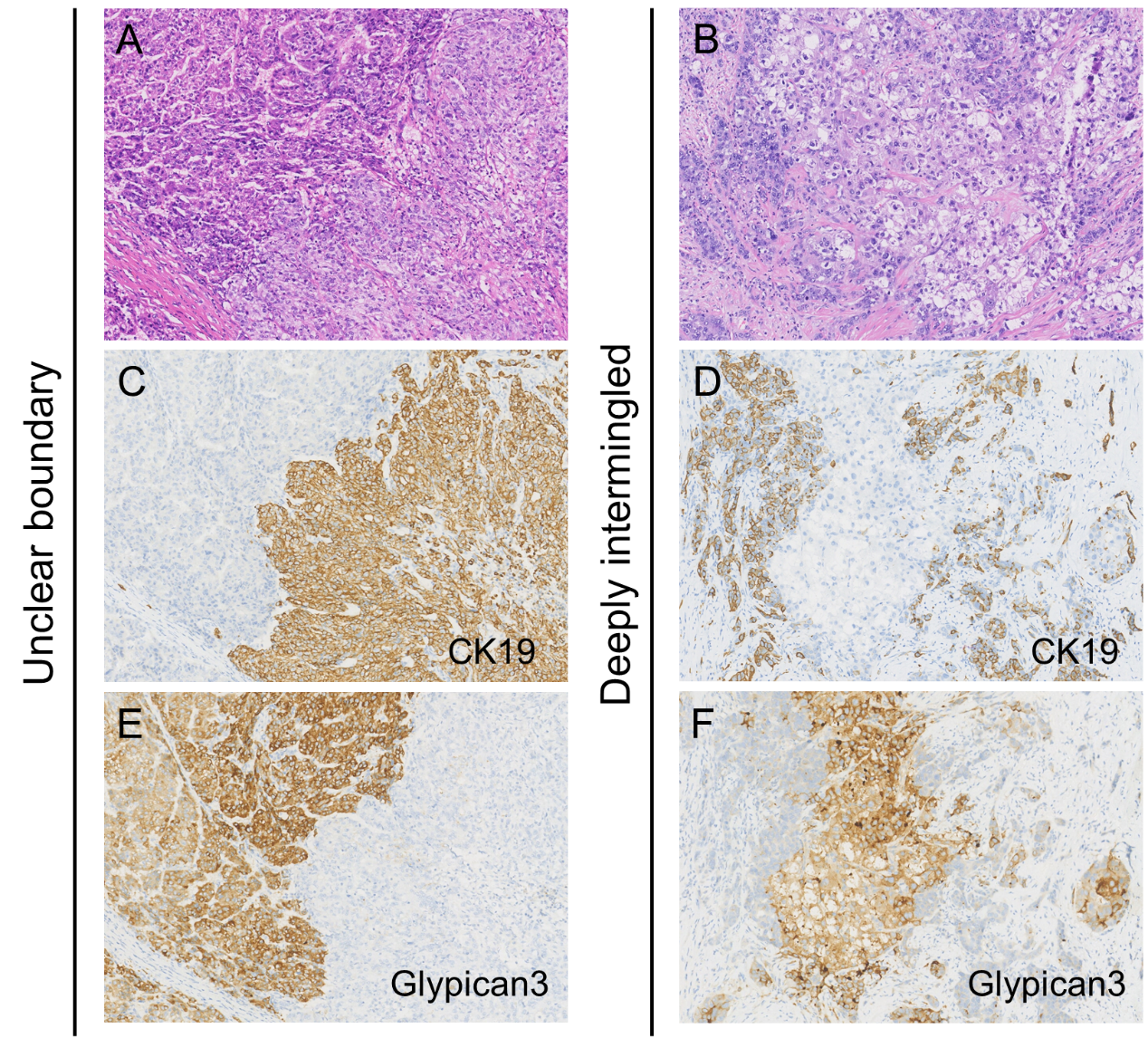


**Figure S2. The other mixing patterns of HCC and CCA components in cHCC-CCA.**

(A) HCC and CCA components mix with unclear boundaries (100×), or deep intermingling patterns (B) (100×). (C, D) IHC staining showed that CK19 was positively expressed in the CCA component (100×). (E, F) IHC staining showed that Glypican-3 was positively expressed in the HCC component (100×).
